# The determinants of willingness to continuously use financial technology among university students: Dataset from a private university in Indonesia

**DOI:** 10.1016/j.dib.2022.108521

**Published:** 2022-08-07

**Authors:** Sumardi, Ummu Salma Al Azizah, Herri Mulyono, Anisa Maulita Suryana

**Affiliations:** Universitas Muhammadiyah Prof. DR. HAMKA, Indonesia

**Keywords:** Willingness, Fintech, Technology, University students, Indonesia

## Abstract

The dataset examines the two perceived benefit and risk factors that continuously influence university students' willingness to use financial technology (Fintech). A non-probability sampling technique was employed to target the study participants. A total of 436 students from a private university in Jakarta, Indonesia, completed a self-administered online questionnaire. The collected quantitative data were screened and analyzed using Partial Least Square Structural Equation Modeling (PLS-SEM). The quantitative analysis result revealed that students' willingness to utilize Fintech continuously is associated with their perceived benefits from such Fintech use. Particularly, students perceived that the benefits of seamless transactions offered by the technology had been the most critical factors that promoted their strong willingness. The data provides new insight related to the university students' use of Fintech for their economic and financial activities. The dataset is also significant for financial technology companies to target and attract more users, particularly from those university students. More importantly, the dataset will be useful for university program development to prepare their students with financial literacy.

**Specifications Table**

**Table:** 

Subject	Business, Management
Specification subject	High Business and Financial Technology
Types of data	Primary data, tables, figures, and excel data
How the data were acquired	The quantitative data were collected using a survey method by distributing a Google form link to the study participants
Data format	Raw Analyzed
Parameters for data collection	The collected data were analyzed to explain the two contributing factors (i.e. perceived benefit and perceived risk) of Indonesian university students' continued willingness to use financial technology. Using a non-probability sampling technique, a total of 436 students of a private university in Indonesia participated in the study.
Description of data collection	The current study adapted a five-point Likert scale survey questionnaire to collect the required data. The questionnaire included 32 items classified into three primary constructs: perceived benefits, perceived risks, and continuance intention. The data were presented in the article included the raw and the analyzed data. Seven tables were developed to describe the analyzed the data covering the respondents’ profiles, descriptive statistics, the reliability and validity of the instrument, and correlation and hypothesis testing.
Data source location	Province: Jakarta Country: Indonesia
Data accessibility	Repository name: Mendeley Data Digital identification number: 10.17632/6ncwmyx6y4.3 Direct link to the data: https://data.mendeley.com/datasets/6ncwmyx6y4/3

## Value of the Data

• The data describe the factors contributing to Indonesian private university students' continued willingness to use financial technology (Fintech).
• The dataset makes it possible for financial technology companies to attract more users around the globe, especially university students in Jakarta, Indonesia.
• This dataset will be useful for university program development and financial technology managers to improve their technology.
• The data present that millennials are more aware of using Fintech for their economic and financial activities.
• The data can be used to test the willingness of university students’ perceptions of Fintech usage in a wider context.

## 1 Data Description

The present article describes the quantitative data used to examine the determinants of Indonesian university students' willingness to use financial technology (Fintech) continuously. Data for the current study were collected using a survey method. The five-point Likert scale survey instrument was developed by adapting three primary constructs of Ryu [1], including perceived benefit (*N* = 12), perceived risk (*N* = 16) and continuance intention to reflect students' willingness to continuously use Fintech (*N* = 4). The perceived benefits also included three main subconstructs such as perceived economic benefit (EB), seamless transaction (ST) and convenience (CV). The other perceived risk construct had three subconstructs (i.e. financial risk (FR), legal risk (LR), security risk (SR) and operational risk (OR)); and Continuance intention (CI). The response for ‘strongly agree’ was scored by 5, ‘agree’ = 4, ‘neutral’ = 3, ‘disagree’ = 2, and ‘strongly disagree’ = 1. The original questionnaire was shown to have an acceptable range of internal consistency (Cronbach's alpha > 0.7). However, the assessment of the survey instrument' internal consistency in the current study was performed on each subconstruct and revealed that most of the constructs possessed a high level of internal consistency (Cronbach's alpha > 0.8), except for the perceived risk and security risk that had a moderate level (Cronbach's alpha > 0.6). Seven tables were developed to describe the analyzed the data covering the respondents’ profiles, descriptive statistics, the reliability and validity of the instrument, and correlation and hypothesis testing.

Tables 1 and 2 below describes the respondent profiles (*N* = 400) and the descriptive statistics.

**Table 1:** Profile and characteristics of respondents (*n* = 400).

Attributes	Characteristic	N	Percentage (%)
Gender	Male	104	26%
	Female	296	74%
Department	Accounting	133	33%
	Management	194	49%
	Islamic economics	45	11%
	D3 accounting	14	4%
	D3 tax	14	4%

**Table 2:** Mean, standard deviation, Skewness, and Kurtosis.

Construct	Item	Mean	Median	Standard Deviation	Excess Kurtosis	Skewness
Perceived benefit	1	3.905	4	0.715	−1.038	0.141
	2	3.9	4	0.696	−0.834	0.094
	3	3.828	4	0.691	−0.909	0.243
Economic benefit	1	3.873	4	0.725	−0.759	0.042
	2	3.54	3	0.767	−0.075	0.265
	3	3.78	4	0.712	−0.79	0.223
Seamless transaction	1	3.708	4	0.743	−0.496	0.237
	2	3.68	4	0.705	−0.688	0.373
	3	3.683	4	0.687	−0.666	0.368
Convenience	1	3.842	4	0.726	−1.007	0.211
	2	3.935	4	0.746	−0.939	−0.003
	3	3.92	4	0.72	−0.972	0.081
Perceived Risk	1	3.502	3	0.791	−0.434	0.312
	2	3.3	3	0.7	0.642	0.386
	3	3.783	4	0.704	−0.816	0.247
Financial risk	1	3.41	3	0.76	−0.1	0.565
	2	3.533	3	0.833	−0.474	0.248
	3	3.417	3	0.695	0.122	0.746
Legal risk	1	3.053	3	0.827	0.311	0.274
	2	3.197	3	0.774	0.436	0.391
	3	3.34	3	0.79	0.267	0.384
	4	3.212	3	0.783	0.494	0.141
Security risk	1	3.68	4	0.87	−0.799	0.008
	2	3.2	3	0.8	0.316	0.329
	3	3.658	4	0.849	−0.446	−0.02
Operational risk	1	3.245	3	0.794	0.773	0.045
	2	3.362	3	0.759	0.355	0.283
	3	3.535	3	0.774	−0.25	0.175

Continuance intention	1	3.68	4	0.719	−0.539	0.201
	2	3.598	3	0.725	−0.587	0.546
	3	3.558	3	0.722	−0.418	0.457
	4	3.775	4	0.748	−0.916	0.249

The total of 400 data were obtained after the screening process of the original 432 Indonesian private university students data. As shown in Table 1 above, majority of the participants were 296 (74%) and 104 (26%) respectively, and many of them came from the management department (*N* = 194, 49%), followed by accounting department (*N* = 133, 33%), Islamic economics department (*N* = 45, 11%), and accounting and taxation vocation (*N* = 14, 4%).The 400 data were then analyzed statistically and the result was shown in Table 2, Table 3, Table 4, Table 5, Table 6, Table 7 below.

The measurement and PLS-SEM model is presented in the following figure:

**Fig. 1:**
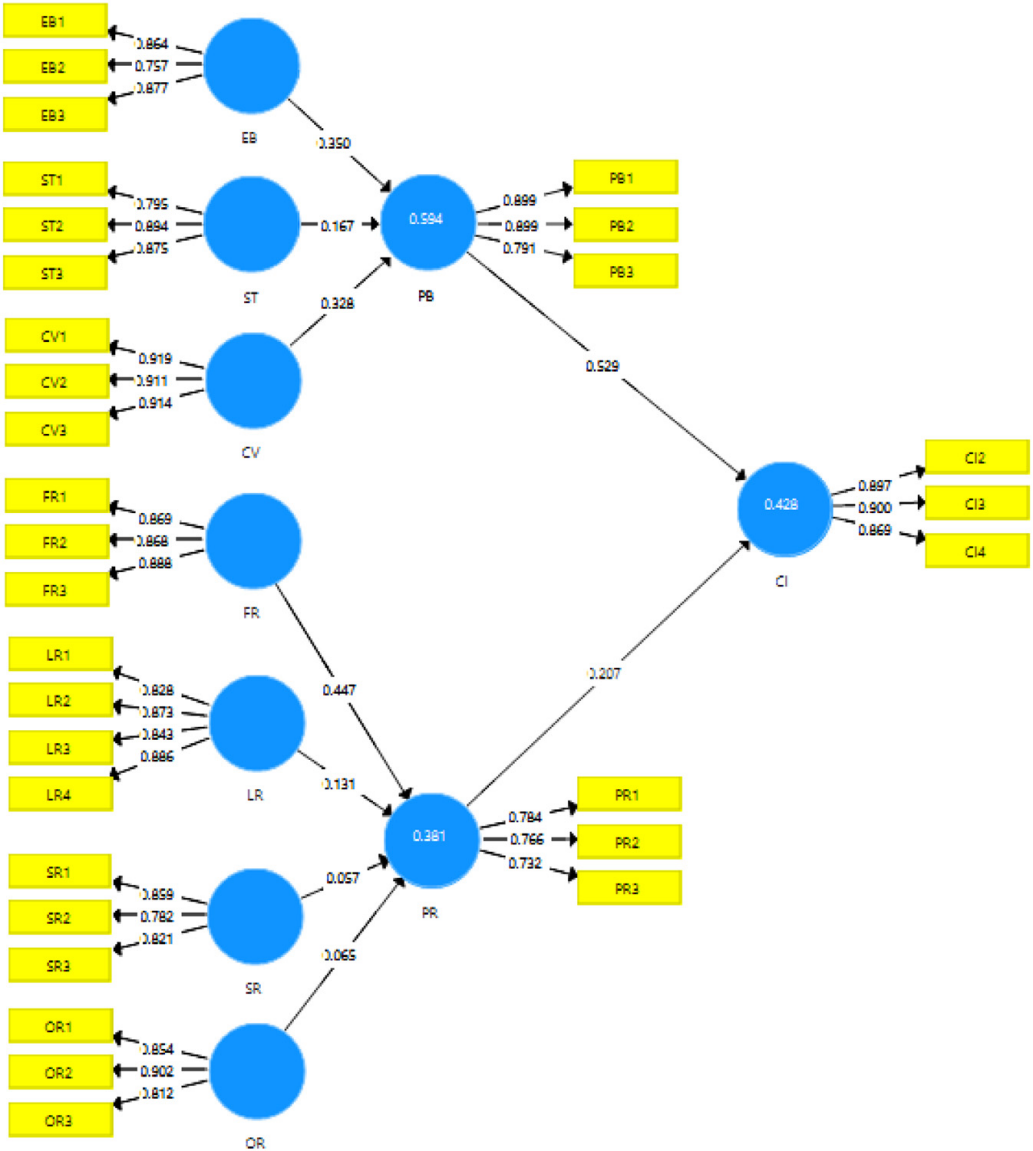
Measurement and structural model analysis.

Tables 3 and 4 describe the reliability and validity of the instrument.

**Table 3:** Reliability and validity.

	Cronbach's alpha (CA)	Rho A	Composite Reliability	Average Variance Extracted (AVE)
Continuance intention	0.867	0.87	0.919	0.79
Convenience	0.902	0.903	0.939	0.837
Economic Benefit	0.784	0.816	0.872	0.696
Financial Risk	0.847	0.849	0.908	0.766
Legal Risk	0.88	0.89	0.917	0.735
Operational Risk	0.817	0.819	0.892	0.734
Perceived Benefit	0.829	0.835	0.898	0.747
Perceived Risk	0.638	0.635	0.805	0.579
Security Risk	0.759	0.76	0.861	0.675
Seamless Transaction	0.817	0.833	0.891	0.732

**Table 4:** Discriminant Validity (Fornell-Larcker criterion).

	CI	CV	EB	FR	LR	OR	PB	PR	SR	ST
CI	0.889									
CV	0.649	0.915								
EB	0.641	0.736	0.834							
FR	0.407	0.478	0.446	0.875						
LR	0.203	0.147	0.192	0.543	0.858					
OR	0.444	0.357	0.36	0.65	0.566	0.857				
PB	0.628	0.703	0.72	0.395	0.14	0.299	0.865			
PR	0.462	0.466	0.576	0.596	0.445	0.467	0.483	0.761		
SR	0.274	0.323	0.292	0.623	0.603	0.653	0.26	0.457	0.821	
ST	0.625	0.698	0.768	0.505	0.256	0.399	0.665	0.573	0.303	0.856

*Root square of AVE.

Table 5 below presents the correlation test and Table 6 shows the hypothesis testing analysis.

**Table 5:** Correlation test.

	Original Sample (O)	Sample Mean (M)	Standard Deviation (STDEV)	T Statistics (|O/STDEV|)
CV -> PB	0.328	0.326	0.048	6.86
EB -> PB	0.35	0.353	0.063	5.579
FR -> PR	0.447	0.445	0.059	7.511
LR -> PR	0.131	0.135	0.062	2.114
OR -> PR	0.065	0.068	0.058	1.114
PB -> CI	0.529	0.529	0.044	11.961
PR -> CI	0.207	0.206	0.05	4.131
SR -> PR	0.057	0.052	0.065	0.886
ST -> PB	0.167	0.168	0.058	2.856

Note. “*ρ* < 0.05.

**Table 6:** Hypothesis testing.

Hypothesis (H)	Path	Original Sample (O)	Sample Mean (M)	Standard Deviation (STDEV)	T Statistics (|O/STDEV|)	*P*-Value	Result
H1	CV -> PB	0.328	0.326	0.048	6.86	0	Supported
H2	EB -> PB	0.35	0.353	0.063	5.579	0	Supported
H3	FR -> PR	0.447	0.445	0.059	7.511	0	Supported
H4	LR -> PR	0.131	0.135	0.062	2.114	0.035	Supported
H5	OR -> PR	0.065	0.068	0.058	1.114	0.266	Not Supported
H6	PB -> CI	0.529	0.529	0.044	11.961	0	Supported
H7	PR -> CI	0.207	0.206	0.05	4.131	0	Supported
H8	SR -> PR	0.057	0.052	0.065	0.886	0.376	Not Supported
H9	ST -> PB	0.167	0.168	0.058	2.856	0.004	Supported

Significant at *ρ* < 0.05 (5%).

The result of coefficient analysis is explained in the Table 7 below:

**Table 7:** The coefficient analysis.

	R Square	R Square Adjusted
CI	0.428	0.425
PB	0.594	0.591
PR	0.381	0.375

## 2 Experimental Design, Materials and Methods

The current data article was part of a study examining the role of benefit and risk factors that continuously influence Indonesian university students' willingness to use financial technology (Fintech). To collect the data for the study, the study questionnaire was distributed online to the target population through a Google form. Using a non-probability sampling technique, a total of 436 data were gathered from a private university in Jakarta, Indonesia; after a screening process, 400 of 436 were analyzed quantitatively. Participants consents were obtained during the data collection process.

The collected data were analyzed using Partial Least Square Structural Equation Modeling (PLS-SEM to gain the best measurement [2,3] and the model is presented in Fig. 1. The collected data were tabulated using an excel application and filtered for missing values and outliers before the analysis. Literature [3,4] has suggested that the number of outliers (residual value higher than 1.96) will be deleted from the data. The removal of outlier data was expected to improve the PLS-SEM results [5]. In addition, the normality of the data was examined by observing the Skewness and Kurtosis. As shown in Table 1, all data corresponded to the acceptable range of Skewness and Kurtosis values. Skewness and Kurtosis values were observed to be normal, showing that Skewness values of the data ranged between 1 and 1, and the Kurtosis values were between 2 and 2. These values indicated that the data were normally distributed.

The reflective measurement for Partial Least Square Structure Equation Model (PLS-SEM) was performed using Smart PLS software. Table 3 below shows the result for the Composite Reliability (CR) and Cronbach's alpha (CA) of all sub-constructs, and Table 4 describes the discriminant validity. A correlation analysis was performed on the data, and the results are shown in Table 4. The results of the correlation suggests that perception was statistically associated with awareness (*r* = 0.840, *ρ* < 0.05) and financial literacy (*r* = 0.885, *ρ* < 0.05). To test the hypotheses presented in this study, the bootstrap technique was employed to calculate the statistical value of t by making a certain number of samples (resampling). The acceptable t values for the two-tailed test were 1.65 (10% significance level), 1.96 (55% significance level), and 2.58 (11% significance level) [2]. The hypothesis testing analysis is shown in Table 6.

Table 6 shows that H1, H2, H3, H4, H6, H7, H9 have a T-Statistic higher than 1.96 with *p* < 0.05. However, H5 and h8 had T-statistics less than 1.96 and *p* > 0.05. Thus, the proposed hypothesis (H1, H2, H3, H4, H6, H7, H9) is supported in this study because it meets the criteria, while the proposed hypothesis (H5 and H8) is not supported. The findings show that the variables CV, EB, and ST significantly affected the PB variable. Furthermore, the FR and LR variables significantly affect the PR variable, while the OR and SR variables have no significant effect on the PR variable. However, it can be seen in Table 5 that the exogenous PB and PR variables have a significant effect on the endogenous CI variable. In addition, the coefficient (β) or path coefficient is also tested for its performance along with the t value. The coefficient (β) shows how strong the influence of a construct is on the other constructs in the structural model. The highest value indicates the most significant influence of the construct as a predictor. Table 5 shows that the highest value is 0.529 for PB, so PB as an exogenous variable has the most significant effect on CI as an endogenous variable.

## Ethical Approval

Ethical approval for the study was obtained from the local ethics commission for social science research, Universitas Muhammadiyah Prof. DR. HAMKA No. 140/F.03.01/2022. Informed consents of all participants had been obtained during the data collection process.

## CRediT authorship contribution statement

**:** Conceptualization, Investigation, Funding acquisition, Supervision, Writing – original draft. **Ummu Salma Al Azizah:** Conceptualization, Investigation, Methodology, Validation, Writing – original draft. **Herri Mulyono:** Conceptualization, Methodology, Data curation, Validation, Supervision, Writing – review & editing. **Anisa Maulita Suryana:** Investigation, Data curation, Project administration, Writing – original draft.

## Declaration of Competing Interest

The authors declare that they have no known competing financial interests or personal relationships that could have appeared to influence the work reported in this chapter.

## References

[1] Ryu H.S. (2018). What makes users willing or hesitant to use Fintech?: the moderating effect of user type. Ind. Manag. Data Syst..

[2] Hair J.F., Ringle C.M., Sarstedt M. (2013). Partial least squares structural equation modeling: rigorous applications, better results and higher acceptance. Long Range Plan..

[3] Al Azizah U.S., Mulyono H. (2020). Dataset on determinants of intention and investment behaviour amongst young Indonesian millennials. Data Brief.

[4] Peng J., Peng S., Hu Y. (2012). Partial least squares and random sample consensus in outlier detection. Anal. Chim. Acta.

[5] Leguina A. (2015). A primer on partial least squares structural equation modeling (PLS-SEM. Int. J. Res. Method Educ..

